# Is obstructive sleep apnoea associated with hypoxaemia and prolonged ICU stay after type A aortic dissection repair? A retrospective study in Chinese population

**DOI:** 10.1186/s12872-021-02226-9

**Published:** 2021-09-06

**Authors:** Xin Xi, Yu Chen, Wei-Guo Ma, Jiang Xie, Yong-Min Liu, Jun-Ming Zhu, Ming Gong, Guang-Fa Zhu, Li-Zhong Sun

**Affiliations:** 1 Sleep Center, Beijing Anzhen Hospital, Capital Medical University, Beijing, China; 2 Department of Respiratory and Critical Medicine, Beijing Anzhen Hospital, Capital Medical University, No. 2 Anzhen Road, Beijing, 100029 China; 3 Department of Cardiovascular Surgery, Beijing Anzhen Hospital, Capital Medical University, Beijing, China

**Keywords:** Obstructive sleep apnoea, Aortic dissection, Hypoxaemia, Intensive care unit

## Abstract

**Background:**

Although obstructive sleep apnoea (OSA) is prevalent among patients with aortic dissection, its prognostic impact is not yet determined in patients undergoing major vascular surgery. We aimed to investigate the association of OSA with hypoxaemia and with prolonged intensive care unit (ICU) stay after type A aortic dissection (TAAD) repair.

**Methods:**

This retrospective study continuously enrolled 83 patients who underwent TAAD repair from January 1 to December 31, 2018. OSA was diagnosed by sleep test and defined as an apnoea hypopnea index (AHI) of ≥ 15/h, while an AHI of > 30/h was defined severe OSA. Hypoxaemia was defined as an oxygenation index (OI) of < 200 mmHg. Prolonged ICU stay referred to an ICU stay of > 72 h. Receiver operating characteristic curve analysis was performed to evaluate the predictive value of postoperative OI for prolonged ICU stay. Multivariate logistic regression was performed to assess the association of OSA with hypoxaemia and prolonged ICU stay.

**Results:**

A total of 41 (49.4%) patients were diagnosed with OSA using the sleep test. Hypoxaemia occurred postoperatively in 56 patients (67.5%). Postoperatively hypoxaemia developed mostly in patients with OSA (52.4% vs. 83.0%, *p* = 0.003), and particularly in those with severe OSA (52.4% vs. 90.5%, *p* = 0.003). The postoperative OI could fairly predict a prolonged ICU stay (area under the receiver-operating characteristic curve, 0.72; 95% confidence intervals [CI] 0.60–0.84; *p* = 0.002). Severe OSA was associated with both postoperative hypoxaemia (odds ratio [OR] 6.65; 95% CI 1.56–46.26, *p* = 0.008) and prolonged ICU stay (OR 5.58; 95% CI 1.54–20.24, *p* = 0.009).

**Conclusions:**

OSA was common in patients with TAAD. Severe OSA was associated with postoperative hypoxaemia and prolonged ICU stay following TAAD repair.

## Background

Aortic dissection (AD) is a lethal disease in which the inner layer of the aorta tears. The Stanford classification divides AD into two groups, namely Type A aortic dissection (TAAD) and Type B aortic dissection. TAAD results from pathological involvement of the ascending aorta and is associated with significant mortality and morbidity despite the numerous apparent improvements in diagnosis and management during the past six decades. Recent data from a study of 4428 patients between 1995 and 2013 show that the in-hospital and surgical mortality rates are still as high as 22% and 18% for patients with TAAD, even with modern medical and surgical/endovascular therapies [1]. Noticeably, for patients with acute [2] or anterograde [3] AD, the fatality remains staggering even following surgical intervention. Therefore, identifying patients at high risk of operative mortality and morbidity will reduce TAAD-related mortality, thus improving its prognosis.

We found previously that hypoxaemia is a common complication in patients with TAAD, and could result in increased postoperative hypoxaemia [4]. Multiple factors could be associated with the development of hypoxaemia following TAAD repair, such as dissection-induced inflammatory response, cardiopulmonary bypass, lung atelectasis, and ischaemic reperfusion injury [4]. Postoperative hypoxaemia prolongs the duration of mechanical ventilation and intensive care unit (ICU) stay, and increases the chances of hospital-acquired pneumonia, reintubation and tracheostomy, and risk of death [4, 5]. Our previous studies showed that more than 30% of patients developed hypoxaemia following TAAD repair and that tracheal reintubation was required in 7.5% of the cases [4, 6–8]. Therefore, identification of the factors associated with postoperative hypoxaemia is essential for optimising treatment and improving patient survival following TAAD repair.

Studies have reported that obstructive sleep apnoea (OSA) is associated with the onset of aortic dissection through arterial dysfunction, hypertension, and augmented negative thoracic pressure [9–11]. Patients with OSA can have poor short-term prognosis as they are at risk of developing hypoxaemia following cardiac and non-cardiac surgeries [12–16]. However, the mechanism by which OSA affects the development of hypoxaemia and prolonged ICU stay in patients undergoing TAAD repair has not been fully elucidated. In this study, we sought to examine: 1) the prevalence of OSA in patients with TAAD, and 2) to evaluate the association of OSA with postoperative hypoxaemia and prolonged ICU stay following TAAD repair.

## Methods

### Study design and patient enrolment

This retrospective study was conducted in 252 consecutive patients with acute or chronic TAAD (either anterograde or retrograde subtype) who underwent total arch replacement between January 1 and December 31, 2018, at the Beijing Anzhen Hospital. TAAD was diagnosed using computed tomographic angiography in all the patients.

Patients would be excluded from this study due to one of the following conditions:

1. diagnosed with OSA and treated by positive airway pressure before surgery,
2. unsuccessful sleep tests owing to clinical concerns, i.e., haemodynamic instability, unbearable pain, severe anxiety, etc.,
3. slept for less than 4 h in the evening of the sleep test owing to insomnia or unbearable pain, and
4. declined to take the sleep test.

**Fig. 1:**
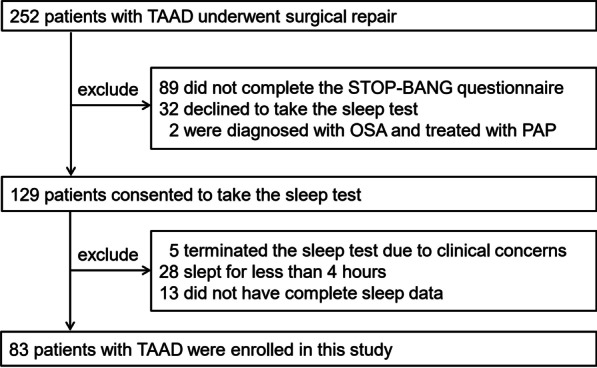
Flow chart of patient enrollment. *TAAD* type A aortic dissection, *OSA* obstructive sleep apnoea, *PAP* positive airway pressure

A total of 124 patients completed the sleep test and 83 of them had complete sleep data and answered the STOP-BANG questionnaire [17]. These 83 patients were included in the final analysis (Fig. 1).

### Sleep study

OSA was diagnosed via completing sleep test within 90 days following surgical procedure. Nox T3 devices (Nox Medical, Reykjavík, Iceland) were used to perform the sleep tests, and an acceptable sleep test should contain a total sleep time > 5 h. Recorded electrodes included nasal pressure transducers, thoracic and abdominal plethysmography, cardiac pulse, snoring, body position, activity, and percutaneous oxygen saturation.

According to the detection of a nasal airflow transducer, apnoea was defined by breath cessation or ≥ 90% airflow drop that lasted longer than 10 s, while hypopnea was verified by at least 30% decline in airflow that lasted longer than 10 s and was accompanied by a 3% decrease in oxygen saturation. Apnoea hypopnea index (AHI) was defined as the sum of apnoea and hypopnea per hour. OSA was diagnosed when the AHI was 15/h or more, and severe OSA was defined by an AHI greater than 30/h. Hypoxaemia during sleep was determined by the average oxygen saturation, the nadir nocturnal oxygen saturation, the percentage of time with oxygen saturation of < 90%, and the oxygen desaturation index (i.e., oxygen saturation drop by ≥ 3% per hour). Daytime sleepiness was calculated using the Epworth Sleepiness Scale (ESS) [18].

Although not accepted as a diagnostic tool, the STOP-BANG score was completed [17] routinely on admission. Patients were assessed by answering “yes” or “no” to eight questions related to major subjective and objective manifestations of OSA. The sum of scores ranged from 0 to 8, and a modified STOP-BANG score of ≥ 4 was considered a high risk of OSA [19]. The consistency of the STOP-BANG score with the objective sleep test was calculated.

### Surgical management

The indications and techniques of TAAD repair in our centre have been described in detail previously [20]. Briefly, it is performed under cardiopulmonary bypass with selective antegrade cerebral perfusion and involves deployment of a stented vascular graft into the descending aorta via hypothermic circulatory arrest, followed by total arch replacement using a four-branched graft.

### Data collection and definitions

The duration of cardiopulmonary bypass, cross-clamp, antegrade cerebral perfusion, and the entire procedure as well as the amount of packed red blood cells transfused during surgery were collected. To evaluate the severity of the patients’ perioperative desaturation, arterial blood was drawn 2 h before and 6 h after the surgical procedure, and the blood gas was assessed on-site promptly.

Preoperative and postoperative oxygenation indices (OIs) were calculated as the ratio of arterial partial oxygen/inspired oxygen fraction at 2 h before and at 6 h after the surgery. Preoperative or postoperative hypoxaemia was defined as an OI of < 200 mmHg [21, 22]. Postoperative complications, a composite endpoint, included sepsis, pulmonary infection, cardiac dysfunction, acute renal failure, re-exploration for bleeding, surgical site infection, stroke, postoperative delirium, paraplegia or paralysis and gastrointestinal bleeding. Systolic blood pressure (BP) of ≥ 130 mmHg or diastolic of ≥ 80 mmHg were regarded as uncontrolled BP [23] Prolonged ICU stay was defined as a ≥ 72 h stay in the ICU for any reason [24].

### Statistical analysis

Normally distributed continuous variables verified by Shapiro–Wilk test are expressed as mean ± standard deviation (SD), and were compared using the Student’s *t* test. Median and interquartile range (IQR) were used for skewed variables, which were compared using the Wilcoxon test. Categorical variables are expressed as a number (percentage), and were compared using the Pearson’s chi-square test or the Fisher’s exact test. The kappa coefficient was calculated to evaluate the agreement between the preoperative STOP-BANG questionnaire score and postoperative sleep test in the diagnosis of OSA. Adjusted odds ratios (OR) and 95% confidence intervals (CI) were estimated from the multivariate logistic model to determine the relationship between the explanatory variables of preoperative hypoxaemia, OSA and AHI stratification, and response variables of postoperative hypoxaemia and prolonged ICU stay. Covariates included in the adjusted model were age, sex, and body mass index (BMI), which were the confounding variables considering their relevance to both OSA and aortic dissection [25, 26]. A receiver-operating characteristic (ROC) curve was constructed to determine the optimal cut-off of postoperative OI that could predict prolonged ICU stay based on the value yielding the best combination of sensitivity and specificity. Statistical analysis was performed using SPSS 18.0 for Windows (SPSS Inc., Chicago, IL), and a two-tailed *p-*value of < 0.05 was considered statistically significant.

## Results

### Baseline characteristics

This study enrolled 83 patients (66 [79.5%] men, 74 [89.2%] acute and 76 [91.6%] anterograde TAAD). Among these, the diagnosis of OSA was confirmed by sleep test in 41 patients (49.4%). Modified STOP-BANG score of ≥ 4 to predict OSA also demonstrated high consistency with the objective sleep test (golden standard) (Kappa = 0.42, *p* < 0.001). Besides having significantly higher scores of ESS and STOP-BANG, patients with OSA were more likely to have uncontrolled hypertension than those without OSA (95.1% vs. 78.6%, *p* = 0.026) (Table 1).

**Table 1:** Comparison of baseline characteristics of enrolled patients grouped by comorbid OSA

Variable	Total (n = 83)	Obstructive sleep apnoea		*p* value
		Yes (n = 41)	No (n = 42)	
Male gender	66 (79.5)	32 (78.0)	34 (81.0)	0.743
Age (y)	47.7 ± 10.8	50.3 ± 9.7	45.1 ± 11.4	0.023
Body mass index (kg/m^2^)	25.8 ± 3.8	26.7 ± 3.6	24.9 ± 3.9	0.035
STOP-BANG score	5.0 (4.0, 6.0)	5.0 (4.0, 6.5)	3.0 (2.0, 4.0)	0.001
STOP-BANG score ≥ 4	53 (63.9)	35 (85.4)	18 (42.9)	< 0.001
Epworth sleepiness scale	7.0 (4.0, 12.0)	10.0 (5.0, 12.0)	4.5 (3.0, 9.8)	0.004
Apnoea hypopnea index (events/h)	13.8 (5.8, 30.2)	30.2 (20.5, 48.3)	5.9 (3.0, 9.4)	< 0.001
Oxygen desaturation index (events/h)	14.3 (5.5, 26.9)	26.0 (19.8, 46.3)	5.9 (2.5, 8.9)	< 0.001
MinSaO_2_ (%)	78.7 ± 11.1	74.4 ± 11.3	82.9 ± 9.2	0.001
MeanSaO_2_ (%)	93.9 ± 2.4	93.1 ± 2.3	94.7 ± 2.2	0.005
T90SaO_2_ (%)	2.0 (0.2, 7.4)	5.2 (1.3, 18.3)	0.35 (0, 2.7)	< 0.001
Marfan syndrome	9 (10.8)	2 (4.9)	7 (21.8)	0.156
Family history of aortic disease	10 (12.0)	4 (9.8)	6 (14.3)	0.738
Uncontrolled hypertension	72 (86.7)	39 (95.1)	33 (78.6)	0.026
Dyslipidemia	23 (27.7)	13 (31.7)	10 (23.8)	0.422
Diabetes mellitus	7 (8.4)	4 (9.8)	3 (7.1)	0.713
Chronic obstructive pulmonary disease	11 (13.3)	8 (19.3)	3 (7.1)	0.097
Coronary heart disease	11 (13.3)	5 (12.2)	6 (14.3)	0.779
Cerebrovascular accident	9 (10.8)	5 (12.2)	4 (9.5)	0.738
Current smoker	55 (66.3)	30 (73.2)	25 (60.0)	0.189
Bicuspid aortic valve	2 (2.4)	0 (0)	2 (4.8)	0.494
Aortic regurgitation*	31 (37.3)	14 (34.1)	17 (40.5)	0.551
Preoperative hypoxaemia	39 (47.0)	20 (48.8)	19 (45.2)	0.746
Left ventricular ejection fraction (%)	62.5 ± 5.2	63.1 ± 6.1	62.0 ± 4.2	0.326
Pericardial effusion†	50 (60.2)	29 (70.7)	21 (46.1)	0.054

Data are expressed as mean ± standard deviation, median (interquartile range) or n (%)

MeanSaO_2_, average oxygen saturation; MinSaO_2_, nadir nocturnal oxygen saturation; T90SaO_2_, percentage of time with saturation lower than 90%

*Including moderate-to-severe aortic regurgitation

^†^Refers to pericardial effusion of > 100 ml

**Fig. 2:**
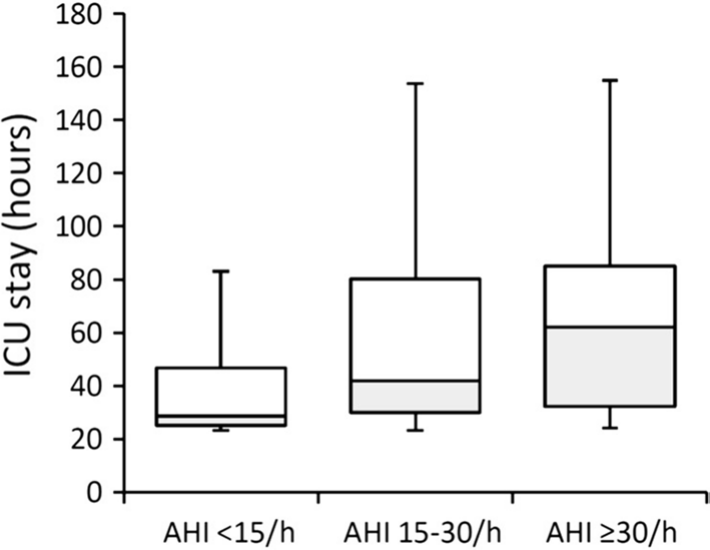
Length of intensive care unit (ICU) stay stratified by apnoea hypopnea index (AHI)

Of the entire cohort, 56 patients developed postoperative hypoxaemia (67.5%) and 22 patients (26.5%) had prolonged ICU stay (> 72 h). Patients with OSA vs. patients without OSA, were more likely to have incidence of postoperative hypoxaemia (83.0% vs. 52.4%, *p* = 0.003) and composite postoperative complications (75.6% vs. 52.4%, *p* = 0.028), and showed significantly longer intubation time (24.6 h vs. 22.4 h, *p* = 0.026) and ICU length of stay (54.0 h vs. 28.5 h, *p* = 0.007) after surgery (Table 2). As shown in Fig. 2, the ICU length of stay was significantly longer in patients with an AHI of > 30/h than in those with an AHI of < 15/h (62.0 h vs. 28.5 h, *p* = 0.008).

**Table 2:** Comparison of operative and postoperative data for enrolled patients grouped by comorbid OSA

Variable	Total (n = 83)	Obstructive sleep apnoea		*p *value
		Yes (n = 41)	No (n = 42)	
*Operative data*				
Cardiopulmonary bypass time (min)	194.2 ± 40.5	194.4 ± 33.0	193.9 ± 47.1	0.951
Cross-clamp time (min)	107.3 ± 28.7	108.1 ± 26.1	106.5 ± 31.3	0.778
Antegrade cerebral perfusion time (min)	25.5 ± 7.9	25.7 ± 8.8	25.4 ± 6.9	0.871
Operative time (h)	7.0 (6.0, 8.0)	7.0 (6.0, 8.0)	7.3 (6.0, 8.0)	0.384
Transfused packed red blood cells (unit)	0.0 (0, 4.0)	2.0 (0.0, 4.0)	0.0 (0, 4.0)	0.398
*Early surgical Outcomes*				
Postoperative hypoxaemia	56 (67.5)	34 (83.0)	22 (52.4)	0.003
Total intubation time (h)	23.3 (20.0, 45.0)	24.6 (21.5, 47.5)	22.4 (19.1, 25.5)	0.026
Length of intensive care unit stay (h)	36.0 (26.0, 75.0)	54.0 (30.0, 87.0)	28.5 (25.0, 52.3)	0.007
Prolonged intensive care unit stay (> 72 h)	22 (26.5)	16 (39.0)	6 (14.3)	0.011
Composite postoperative complications*	53 (63.9)	31 (75.6)	22 (52.4)	0.028

Data are expressed as mean ± SD, median (IQR) or n (%)

*Including sepsis, pulmonary infection, cardiac dysfunction, acute renal failure, re-exploration for bleeding, surgical site infection, stroke, postoperative delirium, paraplegia or paralysis and gastrointestinal bleeding

### Factors associated with postoperative hypoxaemia

Multivariate analysis (adjusted for age, sex, and BMI) showed that preoperative hypoxaemia and OSA were associated with postoperative hypoxaemia. Patients with an AHI > 30/h had significantly higher odds of developing postoperative hypoxaemia vs. patients with an AHI < 15/h (OR 3.28, 95% CI 1.14–10.10, *p* = 0.028) (Table 3).

**Table 3:** Multivariate logistic analyses of risk factors for postoperative hypoxaemia and a prolonged intensive care unit stay

Endpoint/risk factors	Univariate analysis			Multivariate analysis*		
	OR	95% CI	*p *value	OR	95% CI	*p *value
*Postoperative hypoxaemia*						
Preoperative hypoxaemia (yes vs. no)	3.54	1.34–10.29	0.010	3.33	1.16–10.52	0.025
Obstructive sleep apnoea (yes vs. no)	4.42	1.66–12.89	0.003	3.28	1.14–10.10	0.028
AHI > 30/h (vs. < 15/h)	8.64	2.14–58.60	0.001	6.65	1.56–46.26	0.008
*Prolonged intensive care unit stay*						
Preoperative hypoxaemia (yes vs. no)	0.76	0.29–2.05	0.592	0.77	0.26–2.28	0.636
Obstructive sleep apnoea (yes vs. no)	3.84	1.32–11.17	0.010	4.05	1.27–12.90	0.018
AHI > 30/h (vs. < 15/h)	5.46	1.62–18.41	0.005	5.58	1.54–20.24	0.009

*CI* confidence interval, *OR* odds ratio, *AHI* apnoea hypopnea index

*Adjusted for age, gender, and body mass index

### Factors associated with prolonged ICU stay

**Fig. 3:**
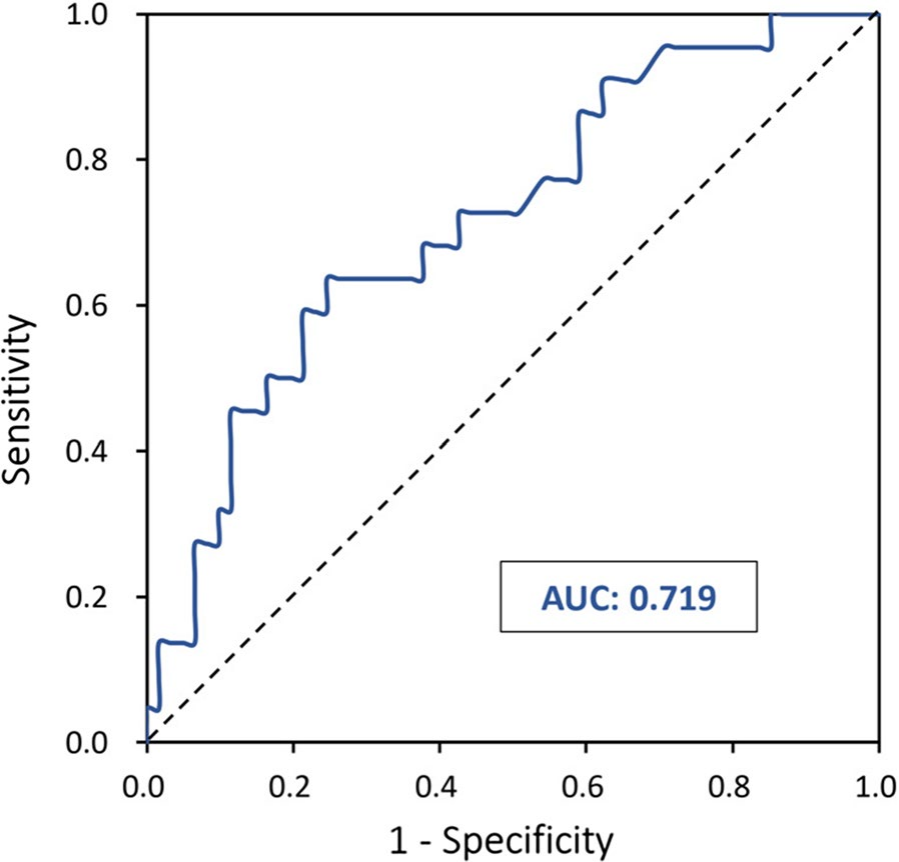
Receiver-operating characteristic curve of postoperative oxygenation index threshold for predicting prolonged intensive care unit stay. *AUC *area under the curve

With an overall area under the curve of 0.72 (95% CI 0.60–0.84; *p* = 0.002) (ROC analysis showed that a postoperative OI of 133.25 was the optimal cut-off value for predicting prolonged ICU stay (sensitivity: 63.6%; specificity: 75.4%; accuracy: 48.3%) (Fig. 3). When taken as a continuous variable, for every unit decrease in postoperative OI, the risk of prolonged ICU stay would be increased by 1% (OR 1.01; 95% CI 1.00–1.02, *p* = 0.008).

OSA was shown to be associated with prolonged ICU stay (unadjusted OR 3.84; 95% CI 1.32–11.17, *p* = 0.010; adjusted OR 4.05; 95% CI 1.27–12.90, *p* = 0.018) (Table 3). Furthermore, compared to an AHI of < 15/h, an AHI of > 30/h (severe OSA) was also identified to be associated with prolonged ICU stay (OR 5.46; 95% CI 1.66–19.49, *p* = 0.005), which persisted following adjustments for confounding variables (OR 5.60; 95% CI 1.59–21.75, *p* = 0.009) (Table 3).

## Discussion

The results of this study show that OSA is prevalent in patients with TAAD, and severe OSA is predictive of postoperative hypoxaemia and prolonged ICU stay following TAAD repair. For patients with TAAD undergoing surgical repair, a sleep apnoea assessment with the STOP-BANG questionnaire prior to surgery could be helpful in recognising individuals who are at high risk of postoperative hypoxaemia and requiring perioperative intervention for OSA.

Here, the high prevalence of OSA in our cohort is in line with previous studies in patients with AD [9, 10]. OSA is considered to increase the risk of AD due to distinctive blood pressure surge and fluctuations, acceleration of atherosclerosis, and uniquely, exaggerated negative thoracic pressure, which induces strong shear forces onto the aorta [27–29]. According to the only literature on the prevalence of TAAD in patients with OSA available as of present [30], middle-aged men with features of being tall, fat and having comorbid hypertension are at high risk of TAAD.

As reported in our previous and present study, many patients with TAAD developed postoperative hypoxaemia, which was closely associated with poor operative outcomes [4–8, 20, 21]. The current study further revealed that OSA was linked to postoperative hypoxaemia, although the underlying mechanisms remain unclear. Upper airway obstruction is less likely to be a reasonable explanation since patients are on mechanical ventilation with tracheal intubation postoperatively. It is not clear whether the postoperative SaO_2_ reduction was caused by pathological responses secondary to OSA, that is, systemic and pulmonary inflammation [31–33], hypercoagulable state, increased oxygen consumption [33, 34], and respiratory muscle fatigue [35]. Memtsoudis et al., found that patients with sleep apnoea had a higher incidence of acute respiratory distress syndrome, a condition of acute hypoxaemia with an OI < 200, postoperatively [15]. Therefore, a sleep assessment before major cardiac surgery may be essential for identifying patients at high risk of developing postoperative hypoxaemia, as shown by our results.

Undiagnosed OSA is an incognitive risk factor for prolonged ICU stay following TAAD repair. Although the mechanism has not been elucidated thoroughly, results from previous studies have indicated that OSA is associated with postoperative complications following cardiac and non-cardiac surgeries [12–14], and this could prolong ICU stay and worsen surgical outcomes. The current study shows that severe OSA predicts postoperative hypoxaemia, which in turn is a strong predictor of prolonged ICU stay. Therefore, identification and treatment of preoperative OSA using non-invasive strategies, such as positive airway pressure could lead to a reduced postoperative hypoxaemia and a shorter ICU stay. Unfortunately, most patients with TAAD are in critical condition and need emergency surgery, which renders the evaluation by preoperative polysomnography, impractical. As intermittent desaturation is mainly caused by breathing events, preoperative oximetry is an alternative test that can be easily performed and used to identify patients with high odds of OSA. In addition, the STOP-BANG questionnaire is another approach to evaluate OSA, which could also serve as a valuable diagnostic clue considering the high consistency between the STOP-BANG score and the results of the sleep test, as shown in our study.

The major limitation of this study is inherent in the nature of TAAD, a clinical catastrophe that has to be managed by an emergency surgery, and this precludes the possibility of having a sleep test before surgical repair. Postoperative sleep assessment together with preoperative questionnaire could still generate incompetent data for determining a preoperative sleep status in individuals with significant changes in body weight and cardiopulmonary function, following an aortic repair. Second, many factors other than OSA can lead to postoperative hypoxaemia and prolonged ICU stay, such as comorbidities, complexity of the procedure and postoperative management. To avoid the risk of model overfitting in statistics, these data were excluded from the multivariate analysis; thus, our conclusion should be extrapolated with caution. This study only included cohorts that underwent a standardised Sun’s procedure for TAAD repair [20] and received a similar post operational care by the same professionals, ensuring the comparability among patients.

## Conclusions

The results of this study demonstrate that OSA was highly prevalent in patients with TAAD, and this could predict postoperative hypoxaemia and prolonged ICU stay following surgical repair. Preoperative sleep assessment among patients with AD help identify OSA; further studies are warranted to investigate whether the treatment of OSA benefits the cohort.

## Data Availability

The datasets used and analysed during the current study are available from the corresponding author on reasonable request.

## Abbreviations

AD: Aortic dissection
TAAD: Type A aortic dissection
ICU: Intensive care unit
OSA: Obstructive sleep apnoea
AHI: Apnoea hypopnea index
ESS: Epworth Sleepiness Scale
OI: Oxygenation index
BP: Blood pressure
SD: Standard deviation
IQR: Interquartile range
OR: Odds ratio
CI: Confidence interval
BMI: Body mass index
ROC: Receiver-operating characteristics
